# Bioinspired Ultra-Low Adhesive Energy Interface for Continuous 3D Printing: Reducing Curing Induced Adhesion

**DOI:** 10.1155/2018/4795604

**Published:** 2018-12-20

**Authors:** L. Wu, Z. Dong, H. Du, C. Li, N. X. Fang, Y. Song

**Affiliations:** ^1^Key Laboratory of Green Printing, Institute of Chemistry, Chinese Academy of Sciences, Zhongguancun North First Street 2, 100190 Beijing, China; ^2^CAS Key Laboratory of Bio-inspired Materials and Interfacial Sciences, Technical Institute of Physics and Chemistry, Chinese Academy of Sciences, Beijing 100190, China; ^3^Department of Mechanical Engineering, Massachusetts Institute of Technology, Cambridge, MA 02139, USA

## Abstract

Additive manufacturing based on liquid resin curing is one of the most promising methods to construct delicate structures. However, precision and speed are limited by the vertical adhesion of in situ cured resin at the curing interface. To overcome the unavoidable adhesion and to develop a general curing interface, we propose a slippery surface taking inspiration of the peristome surface of the pitcher plant. Such surface shows ultra-low adhesive energy at the curing interface due to the inhibition of the direct contact between the cured resin and the solid surface, which also increases the refilling speed of liquid resin. This ultra-low adhesive energy interface is effective for continuous 3D printing and provides insights into the physical mechanisms in reducing vertical solid-solid interfacial adhesion.

## 1. Introduction

Additive manufacturing or three-dimensional (3D) printing rapidly turns computer-aided designs into complex 3D objects on demand. In contrast to conventional manufacturing, additive manufacturing promises to consume less materials and eliminate the use of expensive molds, dies, or lithographic masks [1]. The rapid design and fabrication of soft materials with tunable mechanical [2–4], electrical [5, 6], and other functional properties [7–9] are driving innovations in myriad applications such as biomimetic simulations [10–12], shape-morphing systems [13–15], and soft sensors and robotics [16–19]. Among 3D printing, Digital Light Processing (DLP) is considered as one of the most promising technologies for scalable process as it can allow each slice of 3D model to be solidified at the curing interface simultaneously by using a digitally projected illumination pattern [20, 21]. According to the UV projection direction, DLP 3D printing can be divided into top-down DLP 3D printing, where the curing process occurs at the top liquid resin-air interface, and the bottom-up 3D printing with liquid resin curing at the liquid-solid interface at the vat bottom. In a top-down 3D printing process, the supporting plate is driven downwards with the cured model kept immersing in the liquid resin. The liquid resin depth should be larger than the height of the printed structure, which needs large amounts of liquid resins to be filled. In addition, curing occurs at the liquid-air interface, which is easily influenced by the surrounding environment. Thus, the bottom-up configuration is more widely used. Even though they are effective in constructing high resolution structures in X-Y plane, such bottom-up DLP based additive manufacturing methods still have to overcome several critical challenges, such as limited process speed due to repeated pulling up and down process, low Z-axis resolution, and anisotropic mechanical property, which are essentially originated from the adhesion at the curing interface.

Simplifying the lifting process can lead to a much higher fabrication speed. Recently, continuous liquid interface production method is reported [22] through oxygen inhibition at the curing interface. However, as the oxygen inhibition effect is limited to radical polymerization, the choice of resin and exposure conditions is limited. In addition, the introduction of oxygen can lead to hundreds of micrometers or more of poorly cured surface, which is generally considered as a major obstacle for printing 3D structures of the same scale based on free radical polymerization [13, 23]. In fact, the essential limitation of continuous 3D printing is due to the sticking phenomenon between the cured layer and the curing interface, which is essentially the vertical solid-solid adhesion that was converted from molecular scale liquid-solid contact in the initial state by* in situ *ultraviolet (UV) curing. Thus, the property of the two-dimensional (2D) interface is crucial for continuous 3D printing process [24, 25]. The motivation of our work is to reveal the intrinsic problem of interfacial adhesion and develop a general 2D curing interface for 3D printing.

In nature, insects ‘aquaplane' on the peristome surface of the pitcher plant due to a slippery water layer [26, 27]. Inspired by the phenomenon, numerous researches have been carried out to prepare or investigate slippery surfaces [28–31]. Since the slippery surface could shield the underlying solid substrate from being adhered by other liquid or solid [32, 33], using liquid overlayer to reduce the sticking phenomenon during 3D printing could be an effective solution to existing problems. However, a universal curing interface for arbitrary resins as well as the adhesion mechanism during the 3D printing is a critical challenge. Here, we propose a general curing surface with slippery property, i.e., a slippery ultra-low adhesive surface inspired by the peristome surface, to prevent the* in situ* curing induced adhesion, enhance the resin refilling process, and realize continuous DLP 3D printing. With low vertical adhesion, the cured resin can easily detach from the curing interface. With low horizontal shear, the uncured liquid resin could refill the interface immediately. Complex 3D solid structures with delicate morphologies are drew out of the resin in minutes instead of a few hours with this ultra-low adhesive 2D interface.

## 2. Results

**Figure 1:**
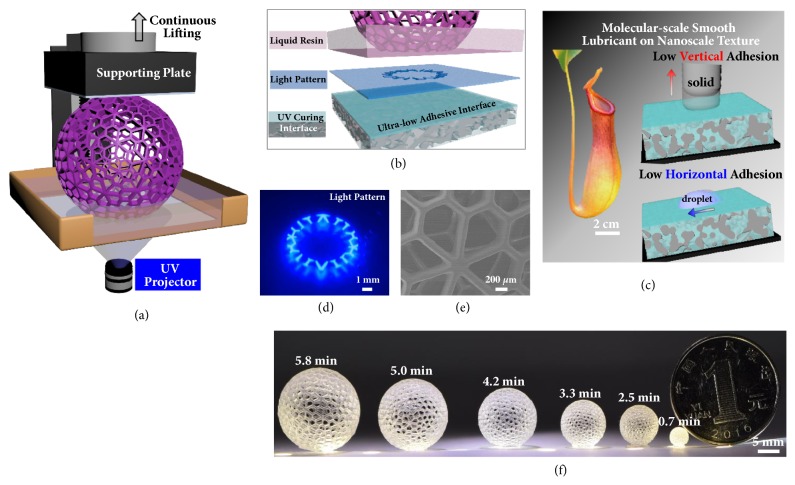
**Continuous 3D construction on the ultra-low adhesive energy interface**. (a) Schematic demonstration of the continuous 3D printing process through simultaneously lifting the supporting plate. Under UV irradiation, the curing of liquid resin continuously occurs at the interface owing to the low adhesive and the sliding property of the curing interface. (b) Scheme of the composition of the investigating system at the curing interface from the side view. Continuous 3D construction at the curing interface is achieved on the ultra-low adhesive energy interface separating the UV light pattern projection plane and the liquid resin. (c) Scheme of the low adhesive surface fabricated from the biomimetic of the peristome surface. (d) Optical capture of the cross-sectional view of UV light pattern at the curing interface during continuous 3D printing. (e) SEM characterization of surface morphology of the 3D printed arts. (f) Optical images of 3D arts fabricated on S-PDMS surface through continuous 3D printing process. The fabrication time presents linear dependence on the model size in the vertical direction, which is independent of the x-y plane.

To test the capability of continuous 3D printing, in our study, we employ a 3D printing configuration with bottom-up UV illumination (Figure 1(a)). Curing interface is modified with a slippery liquid infused layer, and the schematic composition of which is illustrated in Figure 1(b). In detail, inspired by the slippery peristome surface of the pitcher plant, slippery surface with both low vertical and horizontal adhesion is fabricated and used as the UV curing interface for DLP 3D printing (Figure 1(c)). The ultra-low adhesive energy interface used in our experiment is designed based on three criteria: (1) the lubricating liquid must impregnate into, wet, and stably adhere within the polymer network, (2) the polymer network must be preferentially wetted by the lubricating liquid rather than by impinging monomer resin, and (3) the lubricating liquid and the monomer resin must be immiscible. To meet these criteria, in experiment, the cross-linked poly(dimethyl siloxane) (PDMS) is immersed in perfluorocarbon for 24 hours to prepare the ultra-low adhesive energy interface (S-PDMS) as the curing interface. In addition, the curing interface also shows excellent UV transparency and chemical inertness, with little influence on the UV curing process ([Sec supplementary-material-1]). A UV projector is used as the photo-source with the focal plane set at the upper surface of the curing interface [1] (Figure 1(d)).

The S-PDMS surface shows a low adhesive force during the UV curing or printing process ([Sec supplementary-material-1]-[Sec supplementary-material-1]). In detail, the perfluorocarbon functions as a protecting liquid, and it can shield the underlying solid substrate from being adhered by the cured resin [26]. The solid-solid adhesion therefore switches to the solid-liquid-solid adhesion. Even for curing a contact area over cm^2^, the cured resin can detach from the curing interface easily. In addition, this interface enables efficient sliding in the horizontal direction, facilitating the refilling of liquid resin into the newly created interface between the curing interface and the detaching cured resin. Reducing the curing induced adhesion and increasing the resin refilling velocity, continuous 3D printing process with reduced printing time is thus achieved, benefiting from this interface with ultra-low adhesion ability ([Sec supplementary-material-1]).

We tested different combinations of lifting velocities of the supporting plate and light intensities (2.5-43.75 mW/cm^2^) of the UV projector with polyacrylate as the UV curing resin. For example, when resin was cured at a high lifting velocity and a low light intensity, the fabricated structures were elongated or broken during the printing process ([Sec supplementary-material-1]). In contrast, high UV intensity with low lifting velocity would widen the structure. To achieve high construction speed with delicate structures at the same time, the light intensity and switching speed of light patterns should match with the lifting velocity of the supporting plate. Figure 1(f) shows the printed arts with diameters ranging from 4 mm to 20 mm. The printing time of these architectures with layer spacing of 5.0 *μ*m is labeled on each image. For a determined layer slicing thickness and lifting velocity, the fabrication time is in proportion to the printing height. For example, the printing time for a hollow-ball with diameter of 35 mm is about 5.8 min with the printing speed of 100 *μ*m/s, and printing a hollow-ball with diameter of 4 mm needs only 0.7 min. Arrayed hollow-balls with smooth sidewalls (Figure 1(e)) of the same heights (4 mm) can be prepared after the same time ([Sec supplementary-material-1]). This 2D curing interface is therefore facile and effective in constructing 3D models with delicate structures in a fast speed and in a low-cost way.

## 3. Discussion

**Figure 2:**
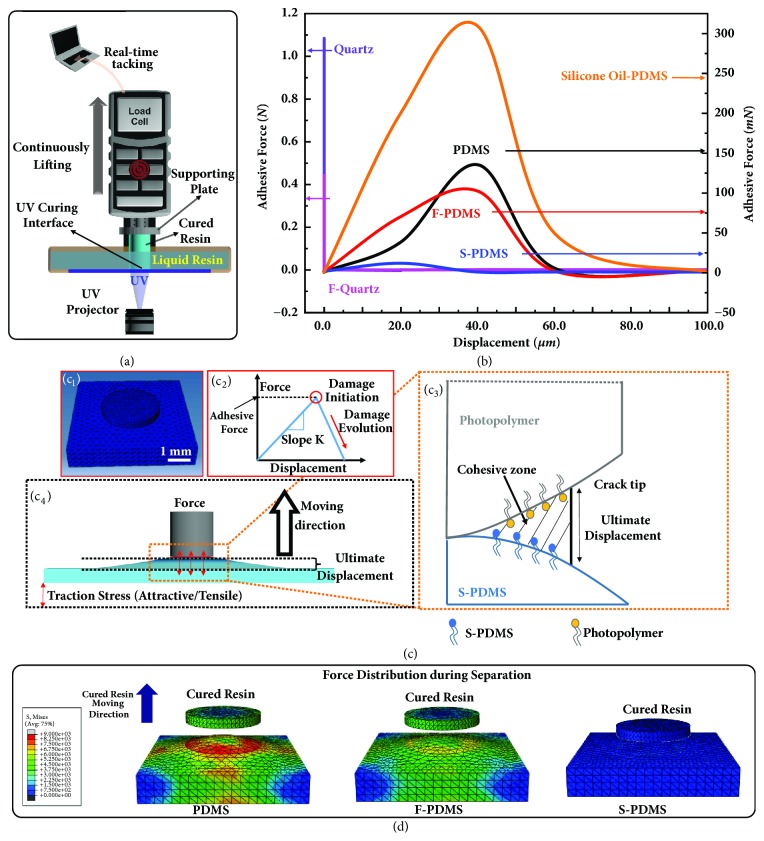
**Comparison and simulation of the separation process and adhesive forces at curing interfaces.** (a) Schematic of the experimental setup for force-displacement curve measurement. The supporting plate is mounted on a load cell with the micro-displacement control stage, which measures the real-time vertical adhesion between the cured resin and the curing interface during the supporting plate lifting process. Elevated by the micro-displacement stage, the cured resin can be peeled off from the resin tank with a steady speed. A circular light pattern is projected onto the curing interface at constant light intensity. (b) Experimental comparison of the adhesive forces between the cured resin and the curing interfaces. Violet, magenta, black, red, orange, and blue lines are force-displacement curves of quartz, F-quartz, PDMS, F-PDMS, silicone oil swelled PDMS, and the ultra-low adhesive S-PDMS interfaces, respectively. The data of quartz-based surfaces use the axis on the left and the data of the PDMS-based surfaces use the axis on the right. (c) Simulation of the separation process between the cured columnar resin and the curing interface. (c_1_) Scheme of the initial stage of simulation, i.e., the contact mode of the columnar resin with the curing interface. (c_2_) Scheme of the bilinear representation of the force-displacement law that represents the Cohesive Zone Model. (c_3_) Scheme of the damage evolution process during the separation process, which results in the deformation of the curing interface. (c_4_) Scheme of the ultimate separation distance where the bonded interfaces are completely separated and interaction force vanishes. (d) Simulation of the force distribution which reveals the detailed separation process of the in situ cured resin from the curing interfaces of PDMS, F-PDMS, and S-PDMS. Compared with PDMS and F-PDMS surfaces, S-PDMS exhibits the lowest adhesive force, the smallest deformation area on the curing interface, and the shortest separation distance, which results in the lowest damage both to the cured resin and to the curing interface.

To quantify the interfacial adhesion during UV curing on different curing interfaces, we conduct* in situ* peel test of the printed units on varied UV-transparent surfaces as illustrated in Figure 2(a) [34]. The supporting plate is mounted on a load cell with simultaneous micro-displacement control, which measures the value of real-time vertical adhesive force between the cured resin and the curing interface with displacement as the variable during the supporting plate lifting process. Elevated by the micro-displacement stage, the cured resin can be peeled off from the curing interface at a steady speed, the process of which is recorded in force-displacement curves. A circular light pattern is projected onto the curing interface at constant intensity: 1 mm diameter is used for quartz-based surfaces, and 5 mm diameter is used for PDMS-based surface, considering the measurement range of the load cell. As controls, we also tested plain quartz surface (quartz), fluorinated quartz surface (F-quartz), and untreated PDMS surface, as well as the fluorinated PDMS (F-PDMS) surface to reveal the critical role of lubricant as the protecting overlayer, which compares the repellence of the surface treated sample with the bulk treated one on the curing induced adhesion. In addition, the commonly used silicone oil swelled PDMS surface is prepared as the control to compare the influence of different protecting overlayers of curing interfaces. First, the adhesion originated from the separation process of a single* in situ *cured layer is measured using the polyacrylate as the test resin system. As shown in Figure 2(b) and [Sec supplementary-material-1]a, the mean adhesive force is the lowest for S-PDMS of 10.0 ± 1.2 mN, followed by F-PDMS of 102.5 ± 3.5 mN, PDMS of 135.5 ± 5.3 mN, and silicone oil swelled PDMS of 309.7 ± 15.6 mN. The vertical adhesive force values for the quartz-based surfaces are much higher with a value of 443.5 ± 7.5 mN for fluorinated quartz and 1084.5 ± 53.1 mN for quartz. It is obvious that S-PDMS outperforms all other tested surfaces in terms of mean adhesive forces for single layer separation. It should be noted that the silicone oil swelled PDMS surface has a large adhesive force due to the miscibility between the monomer of the polyacrylate resin and the silicone oil. For the case of the liquid resin (polyurethane acrylate resin, another typical free radical resin) being immiscible with silicone oil, the adhesive force can be reduced to 6.9 ± 1.8 mN ([Sec supplementary-material-1]). However, the large amount swelling of silicone oil into PDMS will lead to the leaking of oil ([Sec supplementary-material-1]) and pollution to the printed model ([Sec supplementary-material-1]a-[Sec supplementary-material-1]), which impedes its capability as a practical curing interface.

Simulation is conducted from the viewpoint of mechanics in order to interpret the data of the peel test measurement and to understand the effects of interfacial material adhesive properties of curing interfaces on the separation of cured resin from it. The single layer separation process of the cured resin from the curing interface is simulated with Cohesive Zone Model [35, 36], which is carried out in finite element software ABAQUS (ABAQUS, Ver. “6.14 Documentation”; Dassault Systemes Simulia Corporation, 2014). To remain consistent with the experimental condition, a column shaped cured resin is in contact with the top surface of the substrates with no gap at the beginning of simulation (Figure 2(c_1_)). The bilinear traction-displacement law [37] is adopted to provide a schematic view of the separation mechanism (Figure 2(c_2_), [Sec supplementary-material-1]-[Sec supplementary-material-1] in the supplementary information). Once the separation distance reaches the ultimate displacement, the interfaces are completely separated, and interaction force vanishes (Figure 2(c_3_, c_4_)). The model inputs of the heuristic schemes and fitting methods used in simulation are determined through minimizing the error between forces obtained from numerical implementation and experimental measurement [36, 38]. The simulated results indicate that the interface with smaller ultimate displacement and deformation area shows much better performance, which is in well consistence with the experimental results of a single layer separation process (Figure 2(b)). As shown in Figure 2(d) and [Sec supplementary-material-1], comparing with PDMS, F-PDMS, and silicone oil swelled PDMS surfaces, S-PDMS exhibits the lowest adhesive force, the smallest deformation area on the curing interface or the cured resin, and the shortest ultimate displacement, resulting in the lowest damage both to the cured resin and to the curing interface. The successful characterization of the separation process with Cohesive Zone Model confirms the validity of the experimental results and allows for the possibility of predicting and optimizing adhesive forces for more complex geometries. To test the versatility of S-PDMS surface as a curing interface, we measured the mean adhesive force of a typical cationic resin aliphatic epoxy, in addition to the above-mentioned two free radical resins, polyacrylate and polyurethane acrylate. As shown in Figures [Sec supplementary-material-1]b-[Sec supplementary-material-1] and [Sec supplementary-material-1], S-PDMS possesses the lowest value of adhesive force for all tested resin systems, which clearly demonstrates that S-PDMS surface is a general and effective interfacial material for continuous 3D printing process.

**Figure 3:**
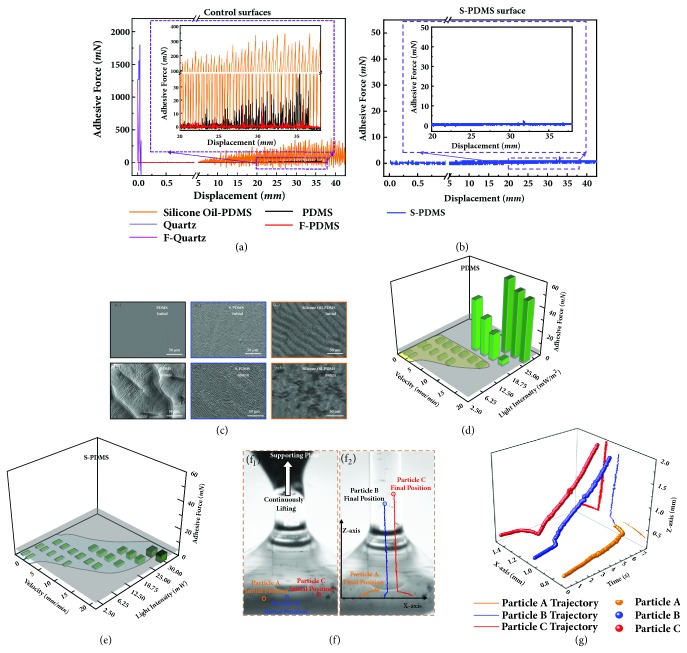
**Real-time measurement of the continuous curing induced adhesion**. (a) Comparison of the real-time force-displacement curves of liquid resin on different curing interfaces during the 3D printing process. Inset is the enlarged part of the force-displacement curve between 20 and 40 mm region for PDMS, F-PDMS, and silicone oil-PDMS surface. Violet, magenta, black, red, and orange lines are force-displacement curves of quartz, F-quartz, PDMS, F-PDMS, and the silicone oil swelled PDMS interfaces, respectively. (b) The real-time force-displacement curves of liquid resin on the ultra-low adhesive S-PDMS curing interfaces during the 3D printing process. Inset is the enlarged part of the force-displacement curve between 20 and 40 mm region with an elevating speed of 10 mm/min. (c) SEM or stereomicroscope images of the PDMS surface, silicone oil swelled PDMS, and the S-PDMS surface used as the curing interface in the initial state and after continuous printing for 40 mm. (c_1_) and (c_2_) are the SEM images of PDMS surface before UV curing and after continuously UV curing for 40 mm, respectively. (c_3_) and (c_4_) are the SEM images of S-PDMS surface before UV curing and after continuously UV curing for 40 mm, respectively. (c_5_) and (c_6_) are the stereomicroscope images of silicone oil swelled PDMS surface before UV curing and after continuously UV curing for 40 mm, respectively. (d) Curing induced adhesion versus lifting velocity of supporting plate and light intensity of UV source on PDMS surface. Grey surface is the indication of the surface where adhesive force is 5 mN. (e) Curing induced adhesion versus lifting velocities of supporting plate and light intensities of UV source on S-PDMS surface. Grey surface is the indication of the surface where adhesive force is 5 mN. (f) and (g) are real-time monitoring of resin refilling process through trace tracking of three nonreactive micro-particles (gold powder, ~100 mesh, Alfa) in the liquid resin during the continuous UV curing process. (f_1_) Initial position of the micro-particles in the liquid resin. Orange, blue, and red circles indicate the initial position of micro-particle A, micro-particle B, and micro-particle C, respectively. (f_2_) Final position of the micro-particles in the liquid resin and cured resin structure. Orange, blue, and red circles indicate the final position of micro-particle A, micro-particle B, and micro-particle C, respectively. (g) The trajectories of three microparticles versus time. Orange, blue, and red lines indicate micro-particle A, micro-particle B, and micro-particle C along with the supporting plate continuously lifting process, respectively.

To investigate the stability and durability of the above-mentioned six curing interfaces, the adhesive force differences of a continuous separation and curing process are investigated through analyzing the real-time force-displacement curves and the morphology evolution of the printed polyacrylate structures during the continuously lifting process of the supporting plate (Figures 3(a)-3(b), with light pattern of 1 mm for all test surfaces). As shown in Figure 3(a) and [Sec supplementary-material-1], the cured resin sticks firmly on the quartz and fluorinated quartz surfaces with vertical adhesive force of ~1750 mN (Figure 3(a), violet line) and ~1000 mN (Figure 3(a), magenta line), respectively. The cured resin breaks at the tip side, leaving a cone structure on the curing interface, due to the large curing induced adhesive force (Figures [Sec supplementary-material-1]b-[Sec supplementary-material-1]c). When the quartz surface is coated by a thin layer of PDMS, F-PDMS, or silicone oil swelled PDMS film, structures can be acquired after the printing process, due to the reduced adhesion between the cured resin and PDMS ([Sec supplementary-material-1]), F-PDMS ([Sec supplementary-material-1]), or silicone oil swelled PDMS (Figures [Sec supplementary-material-1]-[Sec supplementary-material-1]) surface. However, due to continuous curing on such surfaces, adhesive forces become larger and larger, which leads to the appearance of bouncing behavior in the force-displacement curves (Figure 3(a), inset) and pinched structures on sidewalls (Figures [Sec supplementary-material-1]-[Sec supplementary-material-1] and Figures [Sec supplementary-material-1]-[Sec supplementary-material-1]). In comparison, a columnar sample with smooth sidewall can be printed on the S-PDMS surface ([Sec supplementary-material-1]f).

In order to investigate the bouncing behavior in the force-displacement curves for PDMS, F-PDMS, and silicone oil swelled PDMS surfaces, we complement the macroscopic adhesive force results with the analysis of the curing interface morphology and chemical composition by using scanning electron microscope (SEM), stereomicroscope and X-ray photoelectron spectroscope (XPS). For PDMS, cured resin directly drags parts of PDMS substrate away in micro-scales, as the cured resin is in full contact with the curing interface (Figure 3(c) and [Sec supplementary-material-1]). In general, decreasing the surface energy can result in higher repellency [39]. Though F-PDMS has a lower surface energy than PDMS ([Sec supplementary-material-1]), it still cannot prevent the damage of the surface. With the lifting of the supporting plate, much more parts of PDMS (Figures 3(c_1_)-3(c_2_)) are dragged away, which in turn leads to a gradual larger adhesive force and a gradual larger separation distance (Figure 3(a), red line and black line). In addition, due to the intermittent sticking and separation of the cured resin on PDMS, F-PDMS, or silicone oil swelled PDMS surface, pinched structures appear on the sidewall of the printed sample (Figures [Sec supplementary-material-1]d-[Sec supplementary-material-1]e). For S-PDMS, the chemical composition remained unchanged and the surface morphology cannot be damaged by the* in situ *cured resin. Even after an extensive period of printing process over 100 minutes ([Sec supplementary-material-1]), the S-PDMS surface shows very low adhesive force (Figure 3(b)) and almost unchanged morphology both on the S-PMDS surface (Figures 3(c_3_) and 3(c_4_)) and the sidewall of the columnar sample ([Sec supplementary-material-1]f). This behavior is ascribed to the 100-200 *μ*m depth perfluorocarbon infused PDMS (Figures [Sec supplementary-material-1]a-[Sec supplementary-material-1]b), which transfers the high solid-solid adhesion to low solid-liquid-solid adhesion between the cured resin and the curing interface. In detail, the infusion leads to micro-scaled wormlike channels with a geometrical dimension of ~ 3 *μ*m (Figures [Sec supplementary-material-1]c-[Sec supplementary-material-1]d) and nanoscaled holes with a diameter ranging from 10 nm to 400 nm ([Sec supplementary-material-1]e). Considering the dimension of the holes, the pressure for confining the entrapped perfluorocarbon is large enough to stably hold the infused perfluorocarbon without leaking even under severe curing ([Sec supplementary-material-1]). For silicone oil swelled PDMS, when using polyacrylate resin system, the damage of the surface appears due to the miscibility of the silicone oil with the liquid resin, which can be reflected in the force-displacement curve and the sidewall of the cured model, the results of which are similar to the results of PDMS surface (orange line in Figures 3(a) and 3(c_5_)-3(c_6_), [Sec supplementary-material-1]g, and [Sec supplementary-material-1]). When using polyurethane acrylate resin, the adhesive force is low and the force-displacement curve is similar to the S-PDMS surface ([Sec supplementary-material-1]d). However, rough structures still occur on the sidewall of the cured model ([Sec supplementary-material-1]h, [Sec supplementary-material-1]). In a word, the S-PDMS surface with ultra-low adhesive energy demonstrates excellent stability and durability as the curing interface for continuous 3D printing.

The lifting velocity and the light intensity can also influence the morphology of the printed structures ([Sec supplementary-material-1]) and the curing induced adhesion. Taking polyacrylate resin system as an example, in the applicable scope of light intensity and lifting velocity for printing a columnar sample ([Sec supplementary-material-1], green region), the adhesion induced by curing on PDMS (Figure 3(d)), F-PDMS ([Sec supplementary-material-1]a), silicone oil swelled PDMS ([Sec supplementary-material-1]b), and S-PDMS (Figure 3(e)) surfaces increases with the increasing of light intensity and the decreasing of lifting velocity, due to the over-polymerization of single layer caused by larger light input or longer exposure time. For the PDMS-based surfaces, the empirical value of adhesive force is 5 mN with light pattern of 1 mm (grey plane in Figures 3(d) and 3(e)). If the adhesive force is larger than 5 mN, PDMS will be damaged during the curing process. As shown in Figure 3(c), the adhesive force on PDMS surface is smaller than 5 mN when the light intensity and lifting velocity are smaller than 12.5 mW/cm^2^ and 17.5 mm/min, respectively. In comparison, the adhesive force on S-PDMS surface is smaller than 5 mN for all tested light intensities and lifting velocities (Figure 3(e)). Thus, S-PDMS can survive a broad range of lifting velocity and demands little exposure limitations ([Sec supplementary-material-1]).

Besides the low vertical interfacial adhesion, a much stronger refilling ability, is also essential to realize continuous 3D printing ([Sec supplementary-material-1]). The refilling property on the S-PDMS surface during the UV curing process can be directly observed through adding and real-time tracking the locations of nonreactive micro-particles in the liquid resin during the continuous curing process. The X-axis and Z-axis locations of three micro-particles with the variation of time are monitored as shown in Figures 3(f)-3(g) and [Sec supplementary-material-1]. All micro-particles move along with the resin flow into the inner surface of the light pattern and are immobilized in the cured model, which proves the resin refilling property on S-PDMS. In order to measure the refilling velocity of liquid resin on different curing interfaces, a gap is prepared through setting the distance between the cured resin surface and the tested surface as 1 mm. The refilling ability on S-PDMS and other control surfaces are monitored, and the refilling velocities are acquired through tracking the position of the advancing three-phase contact line (TCL) as shown in [Sec supplementary-material-1]. Benefiting from the slippery property, over two times faster resin refilling velocity is achieved on S-PDMS surface than other control surfaces ([Sec supplementary-material-1]). These results parallel the UV curing induced adhesion data indicating that S-PDMS outperforms all the other surfaces in continuous 3D printing.

**Figure 4:**
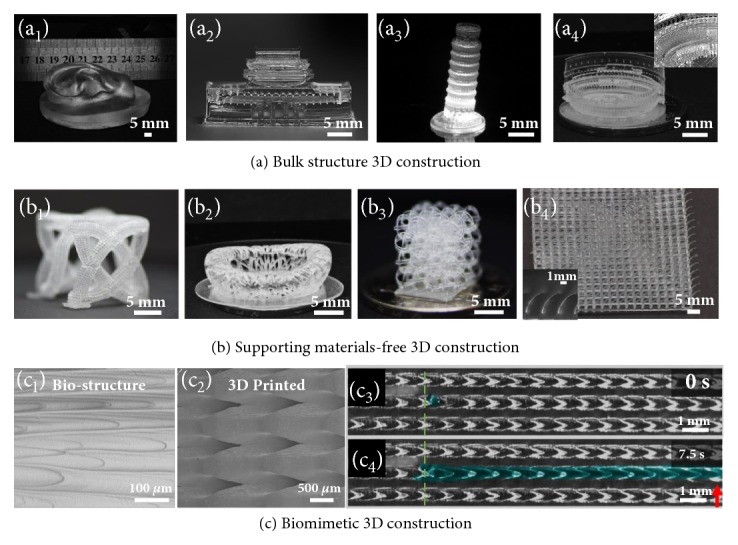
**Rapid fabrication of 3D architectures by continuous 3D printing process. **(a) Optical images of bulk structures including (a_1_) the bulk ear structure with contact area of ~ 7 cm × 3 cm; (a_2_) the gate of Heavenly structure; (a_3_) the Leaning Tower of Pisa structure; (a_4_) the Roman Colosseum structure, fabricated on S-PDMS surface. (b) Complex structures with free standing parts can be fabricated on S-PDMS surface without supporting materials. (b_1_) Optical image of the 3D structure composed of freestanding hollow tubes. (b_2_) Optical image of the bird's nest structure with freestanding inner top edge. (b_3_) Optical image of the 3D structure composed of connecting lines. (b_4_) Optical images of the arrayed bending cilia structures. Inset is the enlarged optical image of the tilt cilia structure. (c) Biomimetic 3D structure fabrication on the S-PDMS surface. (c_1_) SEM image of the structure of the peristome surface of the pitcher plant. (c_2_) SEM images of the biomimetic constructed 3D structure on S-PDMS surface. (c_3_) and (c_4_) are the liquid directional transportation property of the 3D printed biomimetic structure. Green dot line and the red arrow indicate the position of the droplet TCL at 0s and 7.5s, respectively.

Being capable of continuous 3D printing, an array of 3D printed structures is shown in Figure 4, ranging from bulk structures with large cross section (~ 7 cm × 3 cm, Figure 4(a)) to supporting material-free structures (Figure 4(b)) and to biomimetic structures with special wetting property (Figure 4(c)) fabricated on S-PDMS surface. Bulk structures can be fabricated owing to the resin timely refilling property on S-PDMS surface. In addition, fine 3D structures with suspended parts can be achieved without the employment of supporting materials due to the ultra-low adhesion of the curing interface. The first cured suspended parts can be reconnected again with the bulk structure rather than sticking on the curing interface and losing, for example, hollow tube connecting (Figure 4(b_1_), Figures [Sec supplementary-material-1]a-[Sec supplementary-material-1]c), bird's nest model with freestanding inner top edge (Figure 4(b_2_)), line connecting (Figure 4(b_3_), [Sec supplementary-material-1]d-[Sec supplementary-material-1]f), and bending cilia structures (Figure 4(b_4_)). In addition, this strategy can also be extended to biomimetic structure fabrication [40], for example, the structure with directional transportation property [41, 42] (Figure 4(c)), which would promote the wilder applications of 3D printing. Different from previous researches, the fabricating speed is only in relationship with the model height rather than other parameters. This general 2D curing interface opens a broad materials avenue for continuous 3D printing and remarkably increases the refilling ability as well as the utilizing efficiency of materials.

## 4. Materials and Methods

### 4.1. Resins

To measure the vertical adhesive force during UV curing and to fabricate the structures that were subsequently tested, a UV curable resin system was formulated by mixing prepolymer, reactive dilute, photoinitiator, and other additions all tailored to be active at the relevant wave length of UV. Here, three resin systems, polyacrylate system and polyurethane acrylate system that are cured based on the free radical polymerization mechanism and aliphatic epoxy resin that is cured based on the cationic polymerization mechanism, were selected. The polyacrylate system is composed of prepolymer acrylic resin, monomer di(ethylene glycol) ethyl ether acrylate, and photoinitiator 2,4,6-phenylbis (2,4,6-trimethylbenzoyl) phosphine oxide. The polyurethane acrylate system is composed of prepolymer polyurethane acrylate, monomer 2-ethylhexyl acrylate, and photoinitiator 2,4,6-phenylbis (2,4,6-trimethylbenzoyl) phosphine oxide. The aliphatic epoxy resin system is composed of monomer 3,4-epoxycyclohexylmethyl-3,4-epoxycyclohexanecarboxylate and photoinitiator bis[4-(diphenylsulfonio)phenyl]sulfide bis(hexafluoroantimonate) and 4-thiophenyl phenyl diphenyl sulfonium hexafluoroantimonate.

### 4.2. Curing Interfaces

**Quartz** surface was cleaned with acetone, ethanol, and deionized water before use. The** fluorinated quartz** surface was prepared by further fluorination of the quartz interface. The quartz plate was firstly treated with O_2_ plasma (under 70 Pa, 100 mW for 200s). Then it was put into a vacuum dryer, to silanize the quartz surface with 1H,1H,2H,2H-perfluorodecyltrimethoxysilane (PFOS) in a decompression environment at 80°C for 3 hours. After taking out from the dryer, the fluorinated quartz surface is obtained. The** PDMS** prepolymer and curing agent (Sylgard-184 silicone elastomer, Dow Corning, USA) were mixed with a ratio of 10:1, stirred by a mechanical stirrer for 10 minutes, and poured into a self-made vat with a quartz window at the bottom. Then it was put in a vacuum oven to remove bubbles. After putting into an oven heated at 60°C for 4 hours, the PDMS curing interface is acquired. The** fluorinated PDMS** was similarly prepared by O_2_ plasma (under 70 Pa, 100 mW for 100s), silanizing the PDMS surface with PFOS in a decompression environment at 80°C for 3 hours. The** S-PDMS** surface was prepared through adding perfluorocarbon (3M Fluorinert™ FC-3283 or DuPont Krytox GPL105) in the vat and kept in the ambient environment for 24 hours. Before using, the perfluorocarbon is removed and the surface is treated with ethanol, water washing, and nitrogen gas blowing. The S-PDMS surface should be stored in the perfluorocarbon when not in use.

## Supplementary Materials

Supplementary Materials Equipment and Characterization. Supplementary Text. Figure S1. UV-vis absorbance spectra of the quartz, PDMS, and S-PDMS surfaces. Figure S2. The influence of light intensity and lifting velocity on the morphology of the 3D printed structures. Figure S3. Characterization of arrayed hole-balls. Figure S4. The weight ratio of PDMS, silicone oil swelled PDMS, and S-PDMS after violently washing with ethanol. Figure S5. Characterization of the influence of cuing interface on the bottom surface of the cured model for the polyurethane acrylate resin system. Figure S6. Versatility of S-PDMS surface as a continuous printing curing interface. Figure S7. Comparison of the UV curing process on different curing interfaces. (a) Scheme of the experimental configuration. Figure S8. SEM images and XPS characterization of the PDMS surface after continuous curing for different printing distances. Figure S9. Long-term stability of the S-PDMS surface as the curing interface of continuous 3D printing. Figure S10. Detailed characterization of the S-PDMS surface. Figure S11. The influence of miscibility of the lubricant with the liquid resin on the curing induced adhesive behavior. Figure S12. The influence of light intensity and lifting velocity on the morphology of printing a columnar structure. Figure S13. Curing induced adhesion versus the polyacrylate resin system lifting velocity of supporting plate and light intensity of UV source on (a) F-PDMS and (b) silicone oil swelled PDMS surface. Figure S14. Applicable scope of lifting velocity and light intensity for PDMS surface and S-PDMS surface as the curing interface for the polyacrylate resin. Figure S15. The criteria that the substrate should satisfy to realize continuous 3D printing. Figure S16. Refilling time of liquid resin on fluorinated PDMS, PDMS, fluorinated quartz, quartz, and S-PDMS surfaces. Figure S17. Characterization of self-supporting 3D structures fabricated on the S-PDMS surface without the use of supporting materials. Figure S18. Fitted curve of surface tension of quartz surface by OWRK method. Table S1. Wetting property characterization of the silicone oil swelled PDMS surface and the S-PDMS surface. Table S2. Wetting property of three resin systems on the S-PDMS surface. Table S3. Dispersive and polar components of surface energy for the probe liquids. Table S4. Measured contact angles of various liquids on the five used curing interfaces. Table S5. Components of surface energy for the five used curing interfaces. Movie S1. Outline of the ultra-low adhesive energy interface for continuous 3D printing. Movie S2. Simulation of the dynamic separation process of the in situ cured resin from different curing interfaces. Movie S3. Comparison of 3D printing process on silicone oil swelled PDMS and S-PDMS. Movie S4. Real-time tracking of micro-particles in the liquid resin during the continuous UV curing process (10 times acceleration in play speed). Movie S5. Real-time monitoring of the liquid resin refilling process on different curing interfaces.
