# Supplementary material for: Bioinspired Ultra-Low Adhesive Energy Interface for Continuous 3D Printing: Reducing Curing Induced Adhesion
Source: Research (Wash D C). 2018 Dec 20;2018:4795604. doi: 10.1155/2018/4795604 (PMC6750170; doi:10.1155/2018/4795604)
Supplement: Supplementary Materials — Equipment and Characterization. Supplementary Text. Figure S1. UV-vis absorbance spectra of the quartz, PDMS, and S-PDMS surfaces. Figure S2. The influence of light intensity and lifting velocity on the morphology of the 3D printed structures. Figure S3. Characterization of arrayed hole-balls. Figure S4. The weight ratio of PDMS, silicone oil swelled PDMS, and S-PDMS after violently washing with ethanol. Figure S5. Characterization of the influence of cuing interface on the bottom surface of the cured model for the polyurethane acrylate resin system. Figure S6. Versatility of S-PDMS surface as a continuous printing curing interface. Figure S7. Comparison of the UV curing process on different curing interfaces. (a) Scheme of the experimental configuration. Figure S8. SEM images and XPS characterization of the PDMS surface after continuous curing for different printing distances. Figure S9. Long-term stability of the S-PDMS surface as the curing interface of continuous 3D printing. Figure S10. Detailed characterization of the S-PDMS surface. Figure S11. The influence of miscibility of the lubricant with the liquid resin on the curing induced adhesive behavior. Figure S12. The influence of light intensity and lifting velocity on the morphology of printing a columnar structure. Figure S13. Curing induced adhesion versus the polyacrylate resin system lifting velocity of supporting plate and light intensity of UV source on (a) F-PDMS and (b) silicone oil swelled PDMS surface. Figure S14. Applicable scope of lifting velocity and light intensity for PDMS surface and S-PDMS surface as the curing interface for the polyacrylate resin. Figure S15. The criteria that the substrate should satisfy to realize continuous 3D printing. Figure S16. Refilling time of liquid resin on fluorinated PDMS, PDMS, fluorinated quartz, quartz, and S-PDMS surfaces. Figure S17. Characterization of self-supporting 3D structures fabricated on the S-PDMS surface without the use of supporting materials. [file 4795604.f1.zip › 4795604.f1/Supplementary Materials.docx]

Supplementary Materials

**Equipment and Characterization**

Equipment:

**3D Printing Equipment:** The 3D printing equipment is self-made with the composition of LED UV projector (PRO4500, Wintech, China), liquid resin vat (self-made), supporting plate (self-made) mounting on a programmable moving platform (MC600, Zolix Instruments Co., Ltd. China) from bottom to up as displayed in Figure 1a. The UV projector can provide light patterns with projection area of 51.6 mm × 32.2 mm, resolution of 912 × 1140 pixels and light intensity range 0 - 65 mW/cm^2^. The moving velocity range of the programmable moving platform is 1.5 - 100 mm/min with the resolution of 10 μm.

**Model drawing and slicing:** 3Ds Max 2016 Software (Autodesk) is employed to draw the 3D morphology of the models exported with the form of STL. Slicing is conducted through importing the STL file in the software B9Creator with slicing thickness of 5.0 μm. As mentioned in the manuscript, the slicing thickness is 5.0 μm for all printed models to realize a smooth sidewall. After slicing, layer information of the 3D model is exported in the form of BMP image sequences. Images are played sequentially and corresponding light patterns are projected sequentially on the focal plane of the UV projector, which is adjusted as the plane of the upper surface of the curing interface. The play speed of image sequences and the lifting velocity of the supporting plate can be adjusted. The UV light pattern is directly projected on the upper surface of the curing interface without mirrors to adjust the light direction. Layer-by-layer curing is thus realized with appropriate light intensity, play speed of image sequences and Z-axis lifting speed.

**Post-3D printing treatment:** After 3D printing, printed parts were developed in ethanol for 2 min with ultrasonic treatment, then developed in a 1:1 vol/vol solution of methanol and water to remove uncured resin. To enhance the mechanical properties, a post-curing process was performed in a tank with 20 multidirectional LEDs emitting 405 nm light for 30 min at room temperature.

Characterization:

Scanning electron microscope (SEM) images were obtained using a field-emission scanning electron microscope (SEM, JSM-7500F, JEOL, Japan). Stereomicroscope images were captured using a Zeiss Discovery V8 Stereo microscope (Germany). Absorbance spectra was recorded using a fiber optic UV-vis spectrometer (Ocean Optic HR 4000, USA). Contact angles were measured with a contact angle measurement equipment (OCA20, DataPhysics, Germany) with droplets of 3 μL. Each reported contact angle was an average of at least five independent measurements. The surface tension of the liquid resin was measured through a high-sensitivity microelectro-mechanical balance system (DataPhysics DCAT 11, Germany). The adhesive force was measured using the digital load cell (M5-05, Mark-10 Corporation, America), which was mounted on the intelligent moving platform (ESM303, Mark-10 Corporation, America). The threshold force for the load cell is 2.5 N with a force resolution of 0.5 mN and a displacement resolution of 20.0 μm. Force versus distance data was collected by the load cell while supporting plate moving.

**Supplementary Text**

1. Simulation of the separation mechanism between cured resin and the curing interfaces.

In order to interpret the data of peel-test measurement and to understand the effects of interfacial material adhesive properties of curing interfaces on the separation of cured resin from it, simulation is conducted from the viewpoint of mechanics. The single layer separation process of the cured resin with the interface is simulated with Cohesive Zone Model, which utilizes analytical traction-displacement function to characterize the interaction between the used two cohesive surfaces. The implementation is carried out in finite element software ABAQUS, where cured resin with circular cross section is prescribed to be in contact with curing interface before separation initiates. To remain consistent with experimental condition, a column shaped cured resin is placed on top of substrate with no gap at the beginning of simulation. Four edges of the substrate are fixed and a uniform displacement boundary condition is prescribed at the top surface of cured resin to simulate the lifting action. Materials are assumed linearly elastic except for PDMS, F-PDMS and S-PDMS, for which a Neo-Hookean Hyperelastic model is used. At different separation distances, the forces along the vertical lifting direction is integrated over the entire top surface of the columnar cured resin to obtain the macroscopic force, which is the simulated adhesive force.

The most commonly adopted bi-linear representation of traction-separation law can provide a schematic view of the separation mechanism. When three sub-stages, linear elastic regime before damage initiation stage, damage initiation stage and damage evolution stage, are included in the model, the simulated results of traction-displacement curve can fit well with the experimental results. In general, during the separation process, traction first grows monotonically as separation distance increases. The linear elastic behaviour is maintained up to a damage initiation point where certain criterion is satisfied based on material properties. Following the onset of damage, the traction degrades following certain damage evolution principles prescribed. Once the separation distance reaches the ultimate displacement, the bonded interfaces are completely separated and traction vanishes.

In the separation process, the traction vector has three components corresponding to one normal stress and two shear stress components, which are correlated with the displacement vector in the following matrix form. Thus, the sub-stage of linear elastic regime before damage initiation can be expressed as follows:

$\left( \begin{matrix} t_{n} \\ t_{s} \\ t_{t} \end{matrix} \right)=\left[ \begin{matrix} K_{nn} & & \\ & K_{ss} & \\ & & K_{tt} \end{matrix} \right]\left( \begin{matrix} \delta_{n} \\ \delta_{s} \\ \delta_{t} \end{matrix} \right)$ (S1)

$K_{nn}$, $K_{ss}$ and $K_{tt}$ are the contact stiffness in corresponding directions that penalize the separation of bonded interfaces, which can be regarded as three-dimensional counterparts of the slope K in Fig. 2b_2_ in the main text, with the assuming that normal stiffness and tangential stiffness are uncoupled, i.e. all off-diagonal terms in stiffness matrix are zeros. For the damage initiation, we refer to the Quadratic Stress Criterion as the beginning of damage:

$\left( \frac{\left\langle t_{n} \right\rangle}{t_{n}^{0}} \right)^{2}+\left( \frac{t_{s}}{t_{s}^{0}} \right)^{2}+\left( \frac{t_{t}}{t_{t}^{0}} \right)^{2}=1$ (S2)

where parameters with superscript “0” denote the maximal traction in the corresponding directions. The damage evolution is simulated through specifying a scalar damage variable *D*, a monotonic increasing function after damage initiation, and traction evolves according to the following laws:

$t_{i}=\left( 1-D \right)\bar{t}_{i},i=n, s, t$ (S3)

$D=1-\left( \frac{\delta_{m}^{0}}{\delta_{m}^{max}} \right)\left\{ 1-\frac{1-exp\left( -\alpha\left( \frac{\delta_{m}^{max}-\delta_{m}^{0}}{\delta_{m}^{f}-\delta_{m}^{0}} \right) \right)}{1-exp\left( -\alpha\right)} \right\}$ (S4)

2. Calculation of the surface energy of the five used curing interfaces.

The OWRK (Owen, Wendt, Rabel and Kaelble) method.

For interface of solid surface and liquid, work of adhesion can be expressed as follows:

$W_{a}=\gamma_{S}+\gamma_{L}-\gamma_{SL}$ (S5)

The adhesion can be divided into polar and dispersion parts. As proposed by Fowkes, the polar and dispersion interfacial attractions can be treated independently, and the polar-dispersion interactions can be neglected.

$W_{a}=W_{a}^{d}+W_{a}^{p}=2(\sqrt{\gamma_{S}^{d}\gamma_{L}^{d}}+\sqrt{\gamma_{S}^{p}\gamma_{L}^{p}})$ (S6)

According to Young’s equation, when contact angel θ > 0,

$\gamma_{S}=\gamma_{SL}+\gamma_{L}\cos\theta$ (S7)

Combine these equations,

$\frac{\gamma_{L}(1+\cos\theta)}{2\sqrt{\gamma_{L}^{d}}}=\sqrt{\gamma_{S}^{d}}+\sqrt{\gamma_{S}^{p}}\frac{\sqrt{\gamma_{L}^{p}}}{\sqrt{\gamma_{L}^{d}}}$ (S8)

The polar and dispersive components of the solvent is known, thus plotting the left side of Eq. S4 against ${\sqrt{\gamma_{L}^{p}}}/{\sqrt{\gamma_{L}^{d}}}$ will theoretically produce a linear line of data points. Then $\sqrt{\gamma_{S}^{d}}$ and $\sqrt{\gamma_{S}^{p}}$ can be calculated with more than two liquids with given surface tension components. Taking QUARTZ surface as an example, The estimated results are: $\gamma_{S}$ = 42.5 mN/m, $\gamma_{S}^{d}$ = 19.52 mN/m, $\gamma_{S}^{p}$ = 22.93 mN/m as shown in Fig. S18. The polar and dispersive of other interfaces used in this manuscript and the cured resin are calculated from Table S3 and Table S4, and are listed in Table S5.

3. Comparison of the silicone oil swelled PDMS surface and S-PDMS surface.

Corresponding property of the silicone oil (5 cSt, Sigma-Aldrich) swelled PDMS is measured for comparison with the S-PDMS surface, as shown in Table S1. Due to the potential miscibility of silicone oil with the resin system, the property of two kinds of resin (polyacrylate resin and polyurethane acrylate resin) are tested on the S-PDMS surface and silicone oil swelled PDMS surface.

**Table S1**. Wetting property characterization of the silicone oil swelled PDMS surface and the S-PDMS surface.

| **Property** | **Silicone Oil Swelled PDMS** | | **S-PDMS** | |
| --- | --- | --- | --- | --- |
|  | **Polyacrylate Resin** | **Polyurethane Acrylate Resin** | **Polyacrylate Resin** | **Polyurethane Acrylate Resin** |
| Swelling Ratio (wt%) | 67.3 ± 1.3 | | 3.6 ± 0.4 | |
| Swelling Ratio (V%) | 80.5 ± 1.5 | | 0 | |
| CA (°) | 31.1 ± 3.2 | 52.1 ± 2.4 | 56.2 ± 1.4 | 49.2 ± 1.1 |
| Advancing CA (°) | 36.1± 2.1 | 53.0 ± 1.7 | 58.2 ± 1.6 | 52.7 ± 0.5 |
| Receding CA (°) | 0 | 48.2 ± 1.6 | 51.3 ± 2.7 | 47.5 ± 1.2 |
| CA Hysteresis (°) | 36.1 ± 2.3 | 4.8 ± 1.3 | 6.9 ± 2.1 | 5.2 ± 1.0 |
| Adhesive Force (5 mm light pattern, mN) | 309.7 ± 15.6 | 6.9 ± 1.8 | 10.0 ± 1.2 | 5.3 ± 0.5 |

Note: CA is contact angle. wt% is the ratio calculated by weight, V% is the ratio calculated by volume.


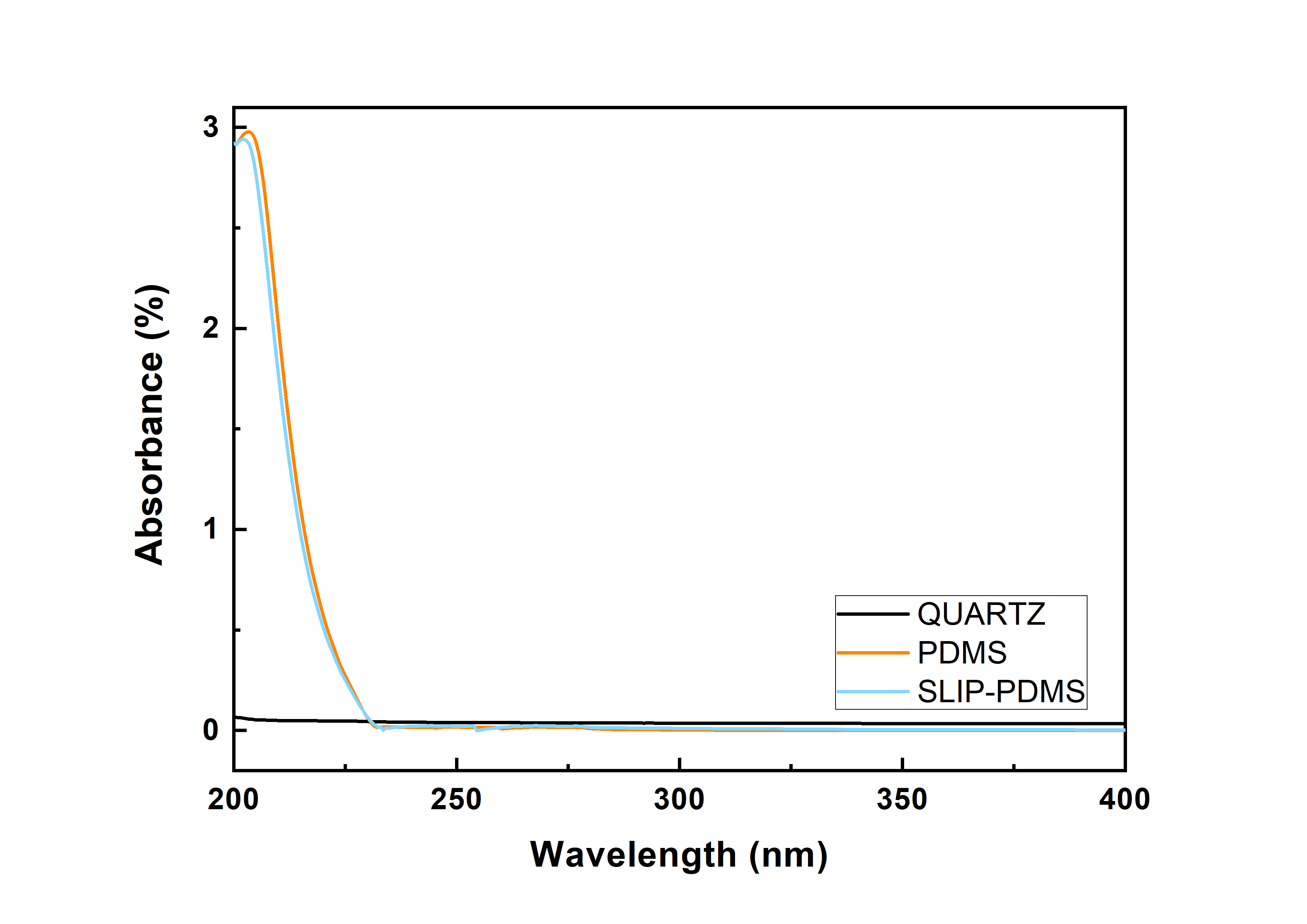


**Fig. S1**. UV-vis absorbance spectra of the quartz, PDMS and S-PDMS surfaces.


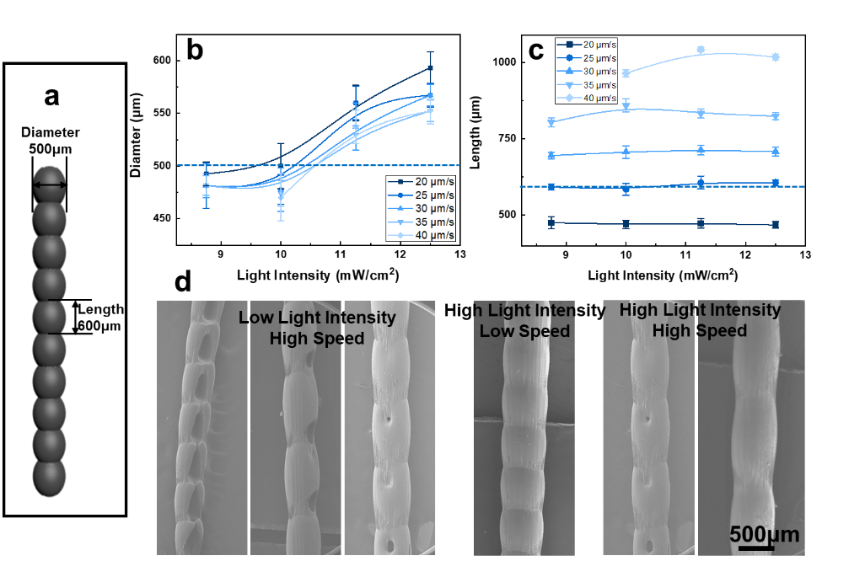


**Fig. S2**. The influence of light intensity and lifting velocity on the morphology of the 3D printed structures. (a) Scheme of the 3D structure used for morphology investigation. The diameter and length of single unit is 500 μm and 600 μm, respectively. (b) The influence of light intensity and lifting velocity on the diameter of the 3D structure unit. (c) The influence of light intensity and lifting velocity on the length of the 3D structure unit. (d) SEM images of examples constructed from different light intensities and velocities.


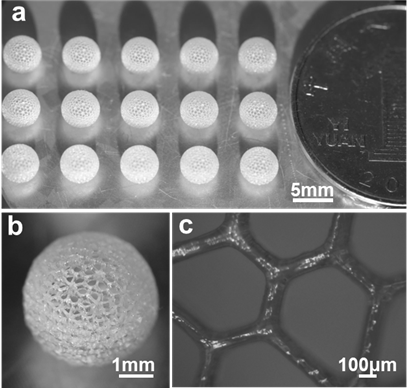


**Fig. S3**. Characterization of arrayed hole-balls. Optical images of arrayed hole-balls (a), single hole-ball (b) and enlarged images of the hole-ball (c). The fabrication time is x-y axis independent and depends on the vertical dimension of the model. Patterned models can be printed in arrays under the same construction time to a single model.


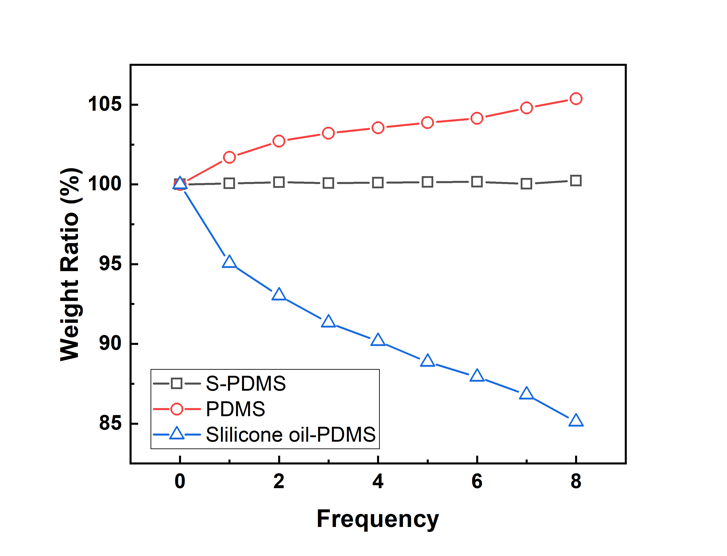


**Fig. S4**. The weight ratio of PDMS, silicone oil swelled PDMS and S-PDMS after violently washing with ethanol. The lubricant reservation ability of S-PDMS surface can also be proved by washing resistance experiment. Ethanol washing is traditionally conducted as it is a good solvent to dissolve uncured resin during the post-processing for the cured printing part with a duration of 10 minutes. Here, we adapt the same way to test the reservation ability for the lubricant with the interval of 10 minutes for each washing process. Through immersing corresponding surfaces into ethanol with magnetic stirring for certain durations, the surfaces are taken out and weighed. The weight ratio (the ratio between the weight after several times’ washing and the initial weight) of different samples are measured. Increasing the immersing times, the weight increases for PDMS and keeps almost the same for S-PDMS. The weight increase of PDMS is due to the swell of ethanol into the PDMS. While weight decreases with the increasing of washing times for the silicone oil swelled PDMS surface, which can be ascribed from the gradual washing away of silicone oil by ethanol. The S-PDMS remained the same weight, which means that ethanol can neither swells the S-PDMS surface nor takes the perfluoro-carbon away, indicating the superior lubricant reserving ability. The immiscibility of perfluoro-carbon with any other liquids also endows S-PDMS surface with stability for a universal DLP 3D printing way to print diverse resins.


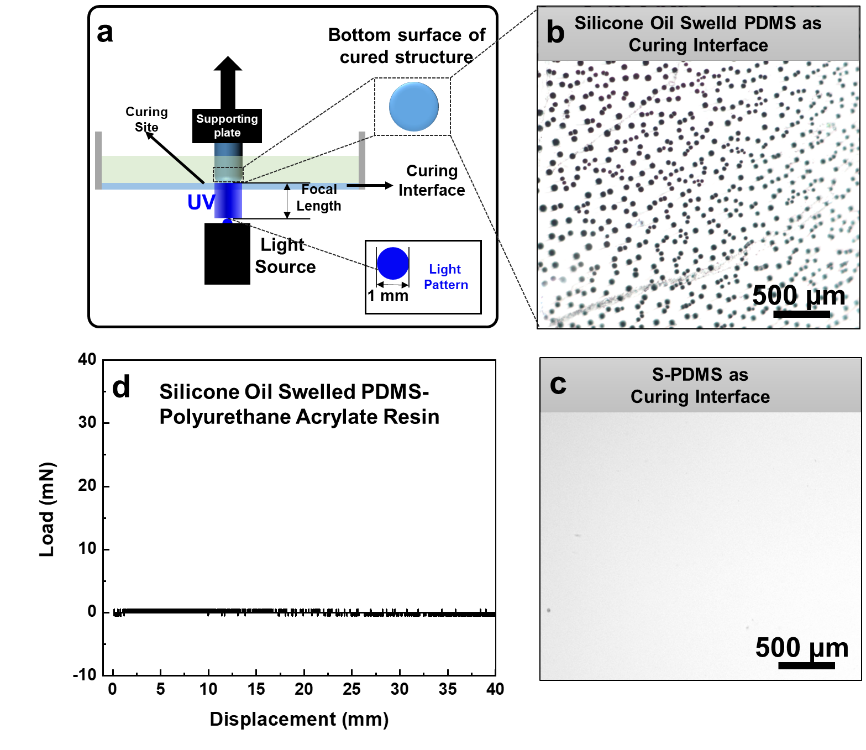


**Fig. S5**. Characterization of the influence of cuing interface on the bottom surface of the cured model for the polyurethane acrylate resin system. (a) Scheme of the experimental configuration. (b) Optical image of the bottom surface of the cured model (polyacrylate resin) using silicone oil swelled PDMS as the curing interface. (c) Optical image of the bottom surface of the cured model (polyacrylate resin) using S-PDMS as the curing interface. (d) Force-displacement curve on silicone oil swelled PDMS surface using polyurethane acrylate resin. The feasibility for S-PDMS and silicone oil swelled PDMS to be used as the curing interface is characterized, where the polyurethane acrylate resin system is immiscible with silicone oil. Even though the silicone oil swelled PDMS has low adhesive force, silicone oil can be taken away and fixed in the cured model. This behaviour not only reduces the amount of the silicone oil, but also leads to pollutions to the printed model. In contrast, for S-PDMS surface, no residual can be found in the cured model.


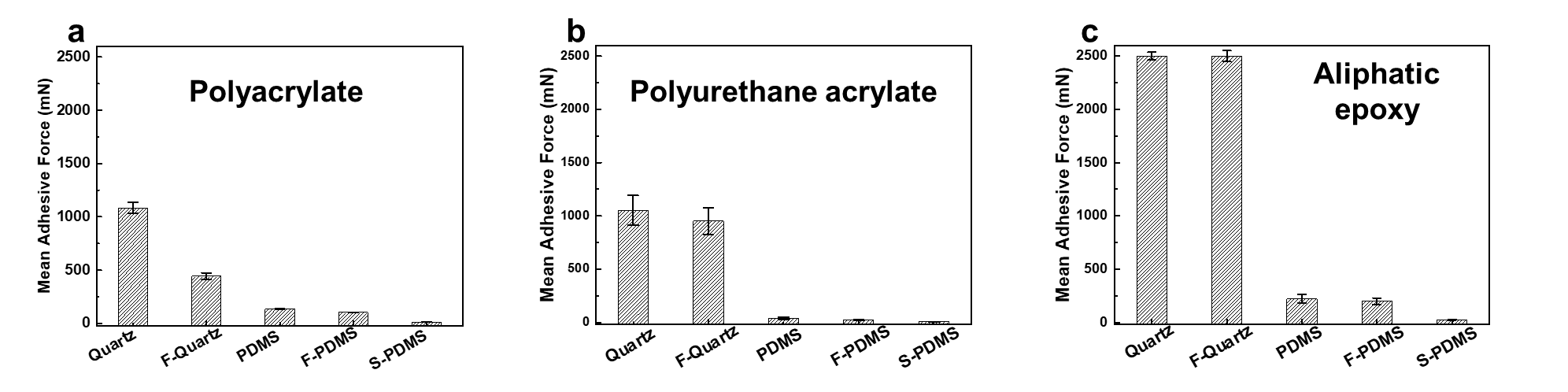


**Fig. S6**. Versatility of S-PDMS surface as a continuous printing curing interface. Mean adhesive forces of three commonly used resin systems, (a), polyacrylate resin, (b), polyurethane acrylate resin and (c) aliphatic epoxy resin on quartz, fluorinated quartz, PDMS, fluorinated PDMS and S-PDMS surface, respectively.


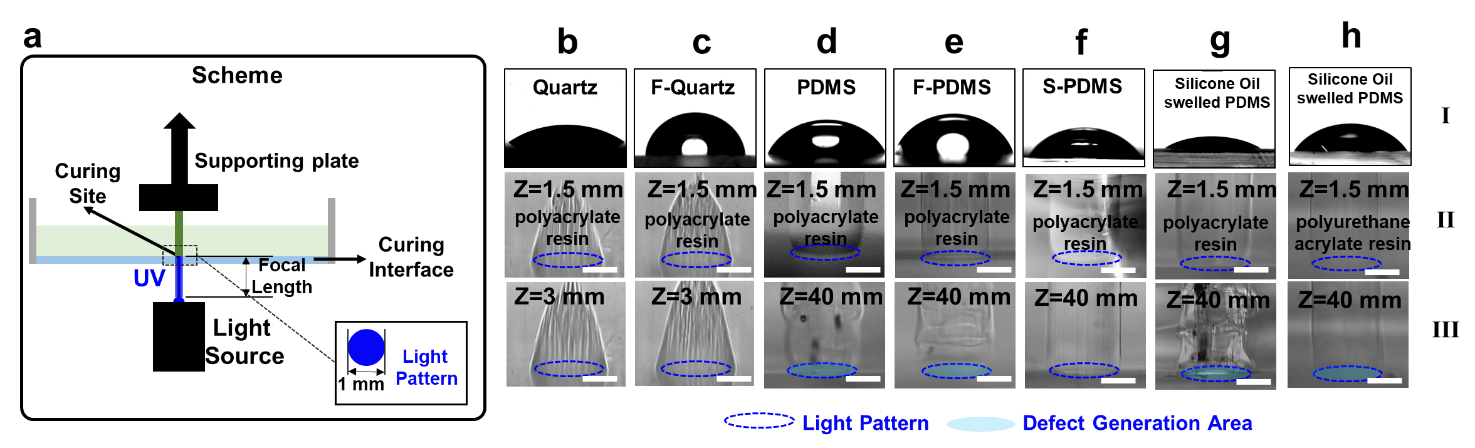


**Fig. S7**. Comparison of the UV curing process on different curing interfaces. (a) Scheme of the experimental configuration. Control experiments on quartz (b), fluorinated quartz (c), PDMS (d), fluorinated PDMS (e), S-PDMS (f) and silicone oil swelled PDMS (g-h) surfaces. (I) and (II) are optical captures of liquid resin contact mode and supporting plate being lifted for 1.5 mm, respectively. (III) is the optical captures of the curing morphology after the supporting plate being lifted for 3 mm for quartz and fluorinated quartz surface, and the curing morphology after supporting plate being lifted for 40 mm for PDMS, fluorinated PDMS, silicone oil swelled PDMS and S-PDMS surface, respectively. The scale bars are 500 μm.


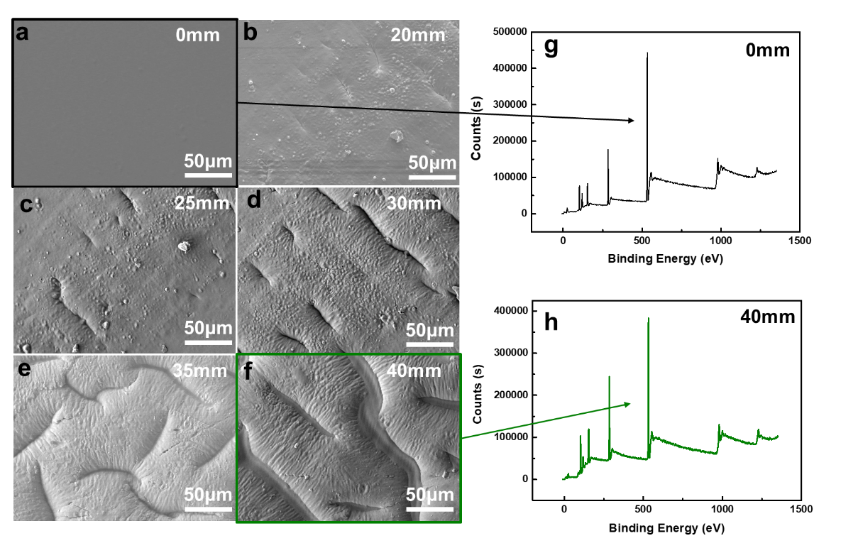


**Fig. S8**. SEM images and XPS characterization of the PDMS surface after continuous curing for different printing distances. SEM images of the PDMS surface in the initial state (a), with printing distances of 20 mm (b), 25 mm (c), 30 mm (d), 35 mm (e), 40 mm (f), respectively. X-ray photoelectron spectroscope (XPS) analysis of the PDMS surface in the initial state (g) and after continuous printing for 40 mm (h), respectively. The PDMS surface changed the surface morphology rather than surface composition after continuous printing. Cured resin directly dragged away part of the PDMS in micro-scale. With the continuous lifting of the supporting plate, much more PDMS is dragged away, which in turn leads to a rougher structure and a larger adhesion.


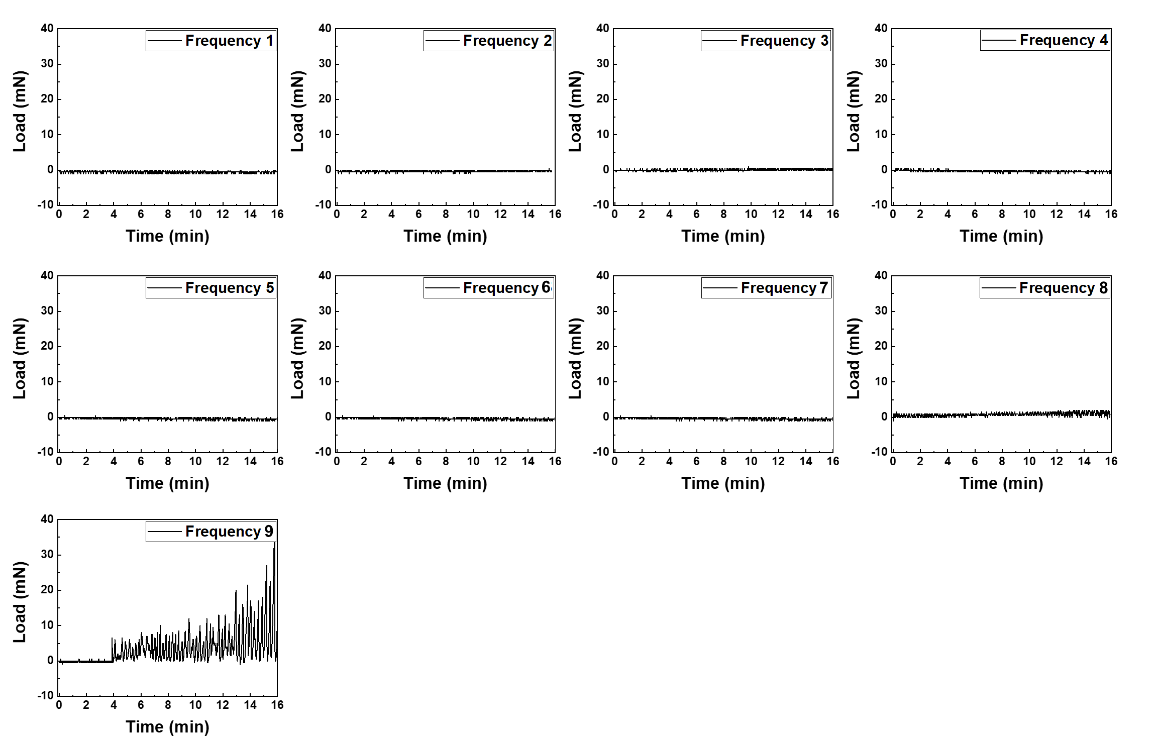


**Fig. S9**. Long-term stability of the S-PDMS surface as the curing interface of continuous 3D printing. The long-term operation is conducted through continous curing on the same area until the force can be detected, as the force of 1 mm circular light pattern on the S-PDMS surface is out of the lowest range of the load cell. Because the distance of the moving platform which mounts the load cell cannot afford a 2-3 hours’ continuous operation, repeating several times of continuous curing on the same area is employed. Detailed information about the repeating process: The initial position of the supporting plate is set as “0”, and after each cycle the moving platform can return back to the “0” position. The lifting speed of the supporting plate is set as 10 mm/min. The light pattern is set as a round circle with diameter of 1 mm. After continous printing for about 16 minutes at the lifting speed of 10 mm/min, the supporting plate stops moving, the printed structure is removed and the supporting plate returns to the “0” position for the next cycle of printing.


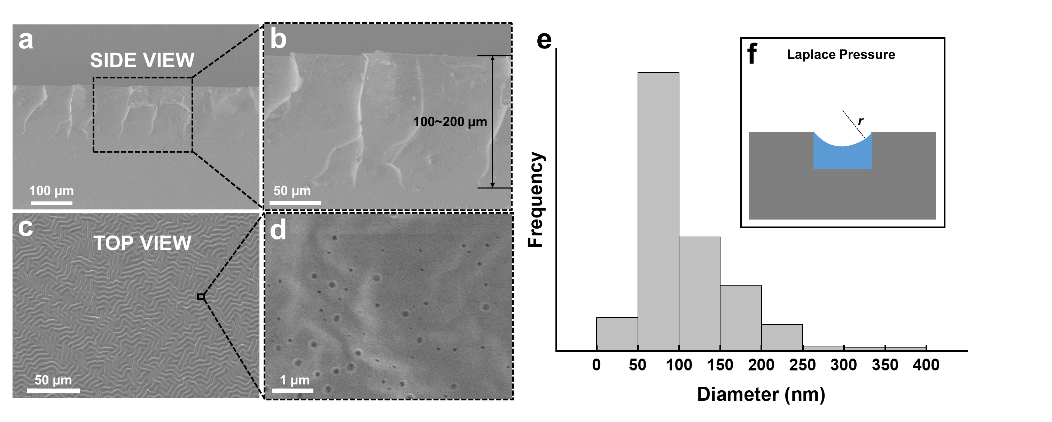


**Fig. S10**. Detailed characterization of the S-PDMS surface. (a) Side view SEM image of the S-PDMS surface. (b) The enlarged view of (a). (c) Top view SEM image of the S-PDMS surface. (d) The enlarged view of (c). (e) The distribution of the hole diameter on the S-PDMS surface. (f) The schematic representation of the Laplace Pressure. The depth of the swelling part is about 100 μm to 200 μm. The slight swell of the PDMS by the perfluoro-carbon will lead to micro-scaled wormlike channels with a geometrical dimension of ~ 3 µm and nano-scaled holes with a diameter ranging from 10 nm to 400 nm. The pressure from the holes and the adhesion during curing are investigated to interpret whether the perfluoro-carbon liquid can be drained out during the curing process. The pressure that the holes can provide to stabilize the perfluoro-carbon liquid is based on the Laplace pressure of the hole structure, *P* = *γ*/*r* (*γ* is the surface tension of the perfluoro-carbon, ~15 mN/m; *r* is the radius of the nano-scaled holes, Fig. R1f)*.* The largest hole (500 nm for example) provides a pressure of ~ 60 KPa to stabilize the perfluoro-carbon liquid. The adhesion between the cured resin and curing interface is only ~ 0.5 Kpa (calculated from the data of Fig. 2d) during the printing process. Thus, with comparatively larger Laplace pressure than the curing induced adhesion, the perfluoro-carbon could be stably confined in structures without being drained out.


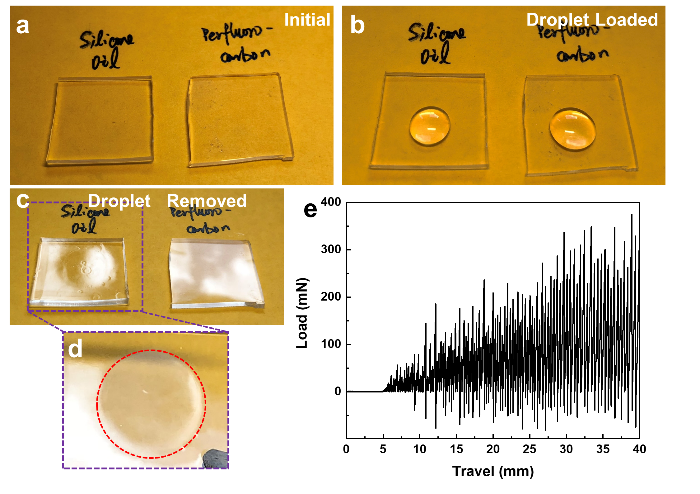


**Figure S11**. The influence of miscibility of the lubricant with the liquid resin on the curing induced adhesive behaviour. (a) Optical image of the initial state of the silicone oil swelled PDMS surface (left) and the perfluoro-carbon infused PDMS surface. (b) Optical image of the resin droplet loaded on both surfaces. (c) Optical image of the surface condition after the resin droplet is removed. (d) Enlarged view of (c) of the silicone oil swelled PDMS. A round shape of trace with the same dimension of the resin droplet is left on the silicone oil swelled PDMS surface. (e) Force-displacement curve of using silicone oil swelled PDMS as the curing interface for continuous curing. For the polyacrylate resin system in our investigation, silicone oil is miscible with the monomer (di(ethylene glycol) ethyletheracrylate). As shown in Fig. S11a-d, an obvious and unrecoverable trace with the similar dimension of droplet is observed when the resin droplet is in contact with the silicone oil swelled PDMS surface. The miscibility of the resin with silicone oil will damage the surface at least on the surface morphology. The curing induced adhesive property of the polyacrylate resin on the silicone oil swelled PDMS surface is also teated. As shown in Fig. S11e, the force measured on the force-displacement curve is much larger than pure PDMS (Fig. 3a in the manuscript) under the same experimental condition. Considering requirements of low adhesion, immiscible with liquid resin and low volume swelling ratio, the S-PDMS surface is selected as the curing interface in our manuscript.


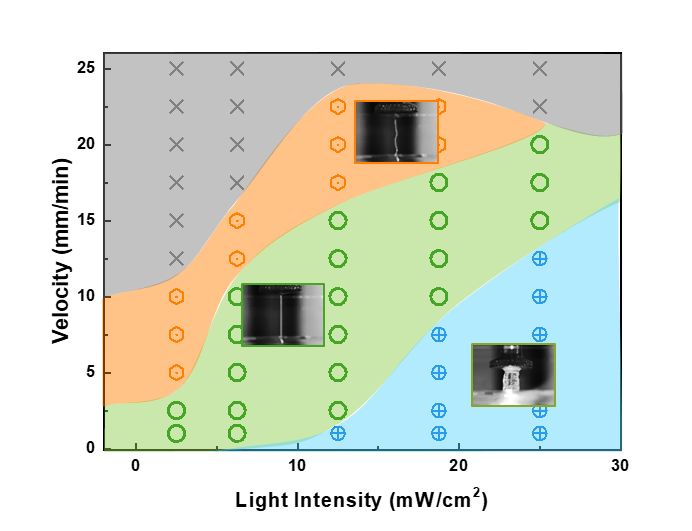


**Fig. S12**. The influence of light intensity and lifting velocity on the morphology of printing a columnar structure. Grey region indicates that columnar structures cannot be acquired. Orange region indicates that curved columnar structures can be acquired with insufficient single layer curing. Green region indicates that columnar structures can be successfully fabricated. Blue region indicates that significantly widen and rough columnar structures are acquired with over-polymerization of single layer.


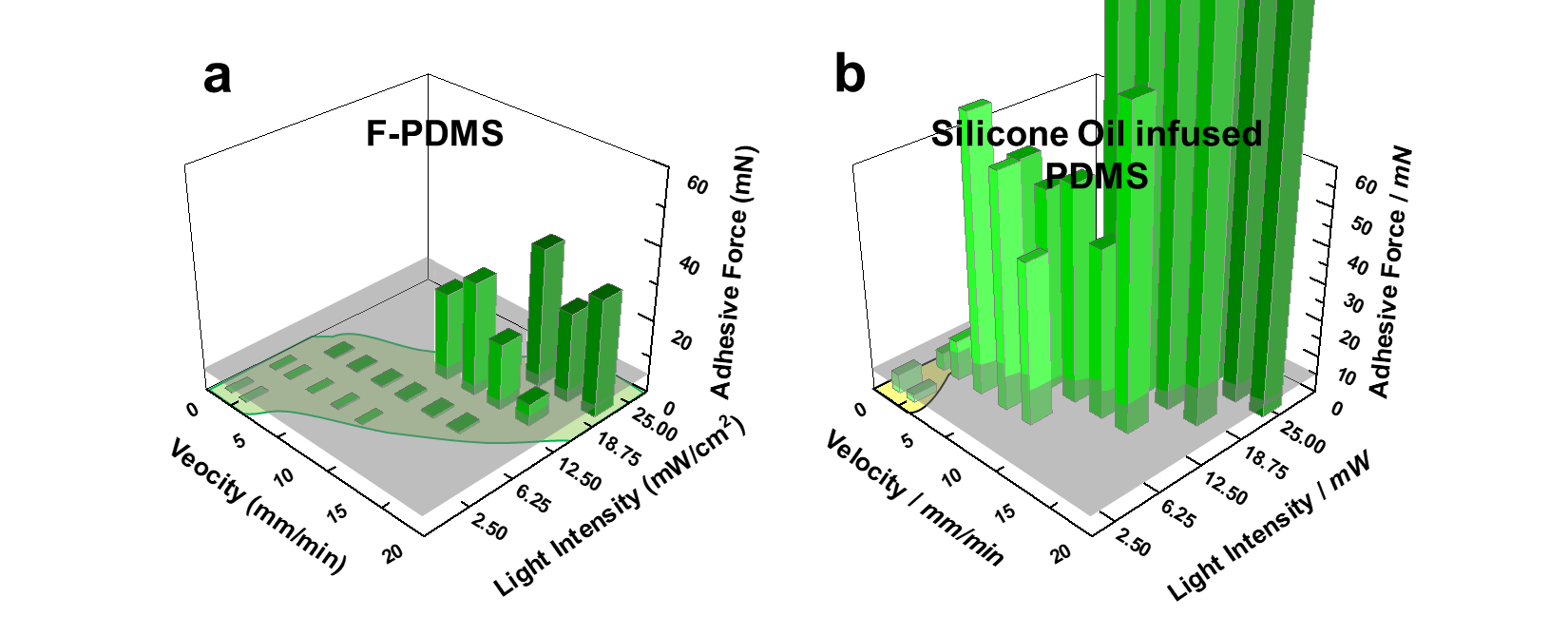


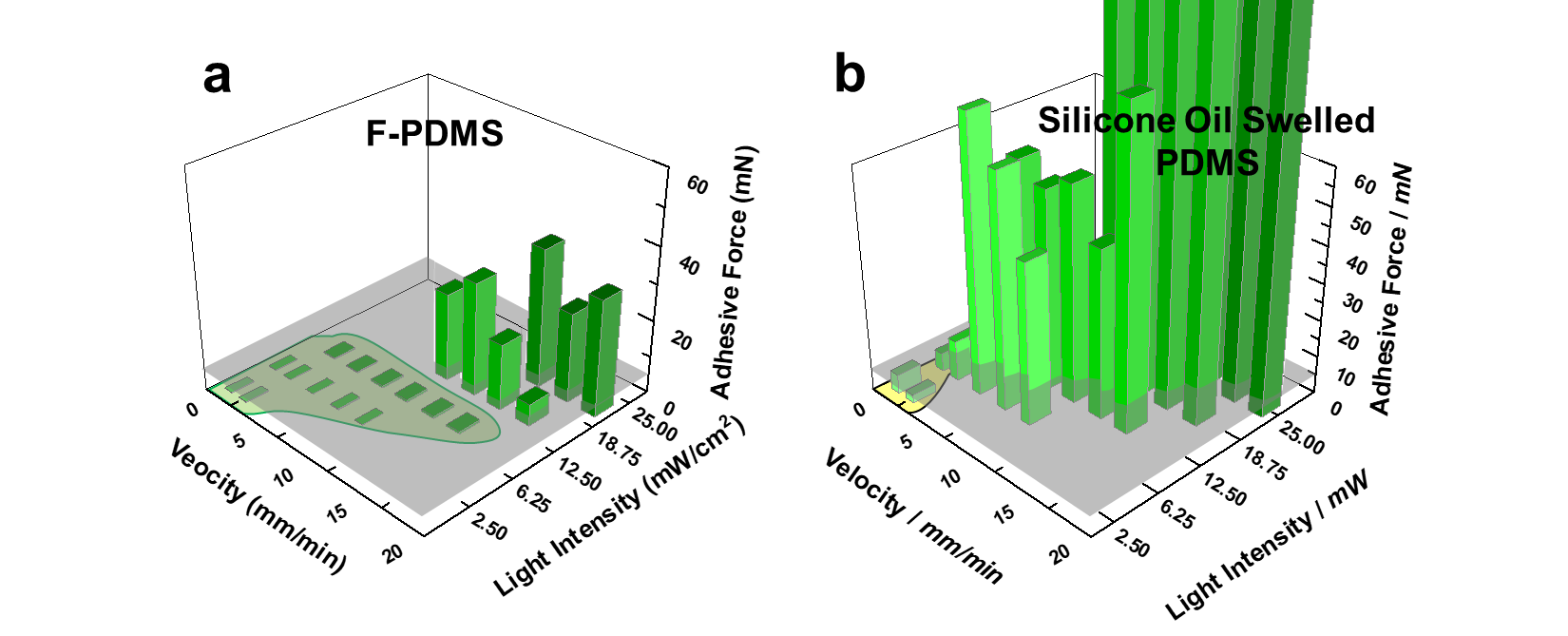


**Fig. S13**. Curing induced adhesion versus the polyacrylate resin system lifting velocity of supporting plate and light intensity of UV source on (a) F-PDMS and (b) silicone oil swelled PDMS surface. Grey surface is the indication of the surface where adhesive for is 5 mN.


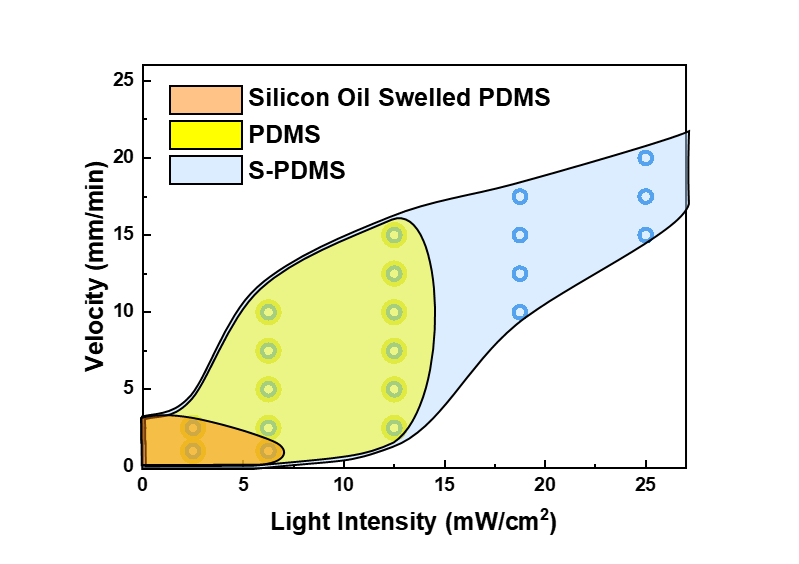


**Fig. S14**. Applicable scope of lifting velocity and light intensity for PDMS surface and S-PDMS surface as the curing interface for the polyacrylate resin. Orange region is the the light intensity and lifting velocity scope of the silicone oil swelled PDMS surface, yellow region is the light intensity and lifting velocity scope of the PDMS surface while light blue region is the scope of the S-PDMS surface.


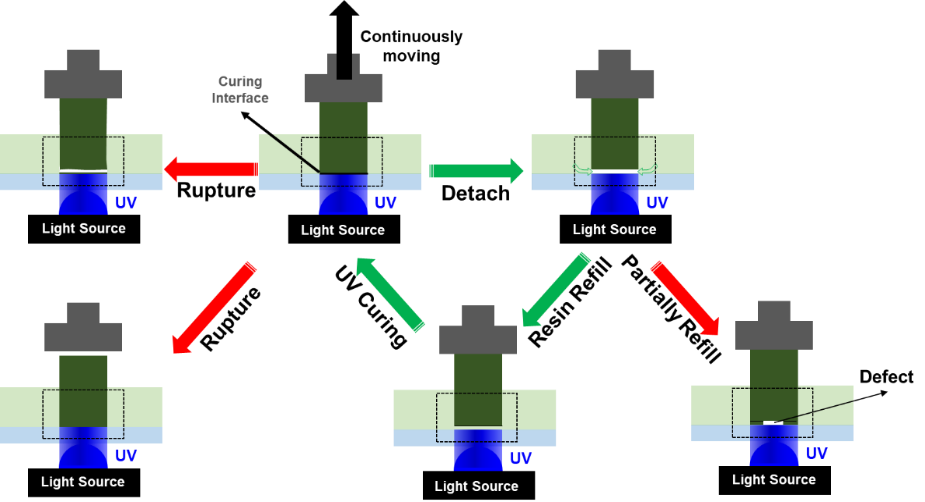


**Fig. S15**. The criteria that the substrate should satisfy to realize continuous 3D printing. In order to realize continuous 3D printing, the curing interface should satisfy two criteria: (1) ultra-low solid-solid adhesion on the curing interface to maintain the timely detaching of the cured resin from the interface and the timely generation of a new curing interface, (2) effective refilling of resin on the newly generated curing interface for successive curing of 3D structure.


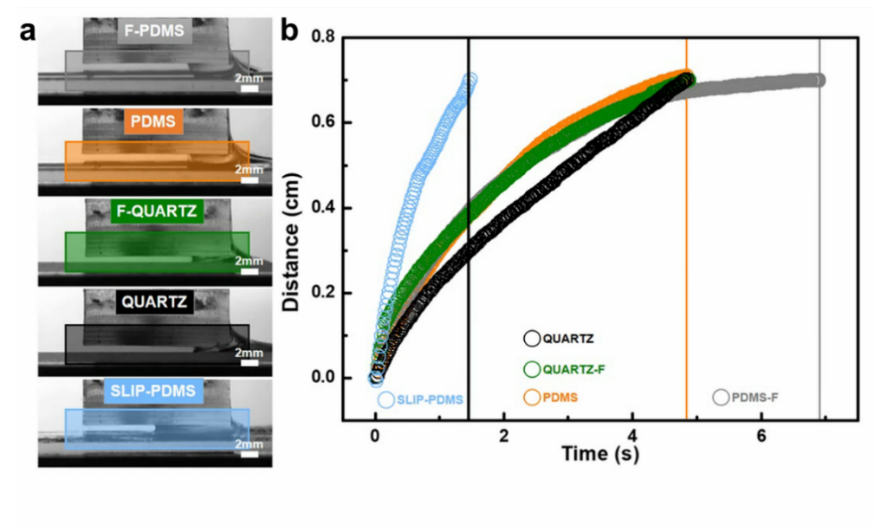


**Fig. S16**. Refilling time of liquid resin on fluorinated PDMS, PDMS, fluorinated quartz, quartz and S-PDMS surfaces. (a) Capture of liquid refilling process when liquid resin refills half-length of the gap. Here, the upper interface is the surface of a solidified liquid resin cuboid, and the lower interface is the five used curing interfaces, the gap between the interface and the solid resin is set as 1 mm. The refilling distance, which is half-length of the gap, is 7 mm. (b) Real time tracking of the front three phase contact lines of the liquid resin on the five used curing interfaces.


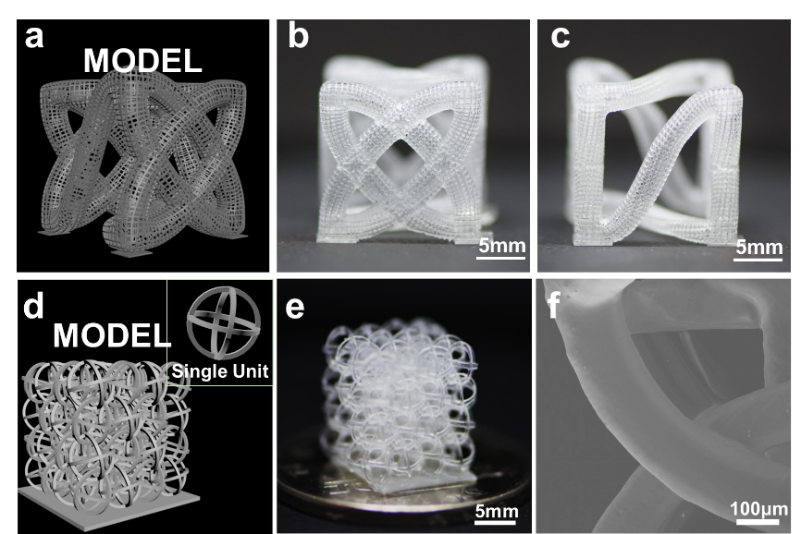


**Fig. S17**. Characterization of self-supporting 3D structures fabricated on the S-PDMS surface without the using of supporting materials. (a), (b) and (c) are the model, and optical images of the 3D structures composed of connecting hollow tubes, respectively. (d), (e) and (f) are the model, optical and enlarged SEM images of the 3D structures composed of connecting lines, respectively.

**Fig. S18**. Fitted curve of surface tension of quartz surface by OWRK method. Five test liquids are used to increase accuracy of the results: water, ethylene glycol, dimethyl sulfoxide, ethylene dichloride and n-hexane. The estimated results are: $\gamma_{S}$ = 42.5 mN/m, $\gamma_{S}^{d}$ = 19.52 mN/m, $\gamma_{S}^{p}$ = 22.93 mN/m.

**Table S2**. Wetting property of three resin systems on the S-PDMS surface.

| **Property** | **Polyurethane Acrylate Resin** | **Polyacrylate Resin** | **Aliphatic Epoxy Resin** |
| --- | --- | --- | --- |
| CA (°) | 49.2 ± 1.1 | 56.2 ± 1.4 | 35.1 ± 1.5 |
| Advancing CA (°) | 52.7 ± 0.5 | 58.2± 1.6 | 38.1± 2.1 |
| Receding CA (°) | 47.5 ± 1.2 | 51.3± 2.1 | 24.7 ± 3.3 |
| CA Hysteresis (°) | 5.2 ± 0.5 | 6.9± 0.6 | 11.4 ± 1.3 |
| Adhesive Force (5 mm light pattern, mN) | 5.3 ± 1.9 | 10.0 ± 1.2 | 19.5± 3.6 |

**Table S3**. Dispersive and polar components of surface energy for the probe liquids.

| Liquid | Abbreviation | SE ($\sqrt{\gamma_{L}}$)  mJ/m^2^ | DSE ($\sqrt{\gamma_{L}^{d}}$)  mJ/m^2^ | PSE ($\sqrt{\gamma_{L}^{p}}$)  mJ/m^2^ |
| --- | --- | --- | --- | --- |
| Water | H_2_O | 72.8 | 21.8 | 51^[1]^ |
| Ethylene glycol | EG | 48.8 | 32.8 | 16^[2]^ |
| Dimethylsulfoxide | DMSO | 44 | 36 | 8^[1]^ |
| Ethylene Dichloride | EDC | 33.3 | 30.8 | 2.5^[1]^ |
| N-hexane | NH | 18.4 | 18.4 | 0^[1]^ |

Note: SE: surface energy, DSE: dispersive component of surface energy, PSE: polar component of surface energy.

**Table S4**. Measured contact angles of various liquids on the five used curing interfaces.

| Liquid | Contact angle (°) | | | | |
| --- | --- | --- | --- | --- | --- |
|  | QUARTZ | QUARTZ-F | PDMS | PDMS-F | S-PDMS |
| H_2_O | 59.9± 2.3 | 106.2±3.3 | 115.1±1.9 | 113.1± 1.2 | 101.9±1.4 |
| EG | 35.9±1.7 | 93.2±2.3 | 103.0±1.1 | 101.7±0.6 | 83.1±1.6 |
| DMSO | 31.9±1.4 | 86.9±1.9 | 94.9±5.1 | 89.3±1.1 | 73.6±2.1 |
| EDC | 14.3±0.6 | 61.1±0.5 | 51.5±1.0 | 71.1±1.4 | 35.8±1.1 |
| NH | 7.4±1.2 | 32.8±4.0 | 7.2±3.7 | 42.2±3.7 | < 5 |

**Table S5**. Components of surface energy for the five used curing interfaces.

| Solid | SE ($\sqrt{\gamma_{L}}$)  mJ/m^2^ | DSE ($\sqrt{\gamma_{L}^{d}}$)  mJ/m^2^ | | PSE ($\sqrt{\gamma_{L}^{p}}$)  mJ/m^2^ |
| --- | --- | --- | --- | --- |
| QURATZ | 42.4 | 19.52 | 22.93 | |
| F-QUARTZ | 15.2 | 14.08 | 1.10 | |
| PDMS | 16.3 | 16.34 | 6.60×10^-4^ | |
| F-PDMS | 12.9 | 12.53 | 0.38 | |
| S-PDMS | 20.9 | 19.76 | 1.11 | |

Note: SE: surface energy, DSE: dispersive component of surface energy, PSE: polar component of surface energy.

**References.**

[1] Van Oss, C. J. Interfacial Forces in Aqueous Media, 2nd ed.; Taylor & Francis: New York, 2006

[2] Mittal, K. L., Ed. Contact Angle, Wettability and Adhesion; VSP:  Utrecht, The Netherlands, 1993.

**Captions of supplementary movies**

**Movie S1**. Outline of the ultra-low adhesive energy interface for continuous 3D printing.

**Movie S2**. Simulation of the dynamic separation process of the *in-situ* cured resin from different curing interfaces.

**Movie S3**. Comparison of 3D printing process on silicone oil swelled PDMS and S-PDMS.

**Movie S4**. Real-time tracking of micro-particles in the liquid resin during the continuous UV curing process. (10 times acceleration in play speed)

**Movie S5**. Real-time monitoring of the liquid resin refilling process on different curing interfaces.
